# Analysis of Codon Usage Bias Between Entomopathogenic Fungus *Ophiocordyceps sinensis* and Its Host, *Thitarodes xiaojinensis*

**DOI:** 10.3390/biology15040346

**Published:** 2026-02-16

**Authors:** Jinxuan Yan, Chuyu Tang, Haoxu Tang, Bing Jia, Chao Feng, Jianzhao Qi, Yuling Li, Xiuzhang Li

**Affiliations:** 1 State Key Laboratory of Plateau Ecology and Agriculture, Qinghai Academy of Animal and Veterinary Sciences, Qinghai University, Xining 810016, China; yanjinxuan2666@163.com (J.Y.); chuyutang0410@163.com (C.T.); haoxutang0717@163.com (H.T.); bingjia0415@163.com (B.J.); 18667661285@163.com (C.F.); 2 Shaanxi Key Laboratory of Natural Products & Chemical Biology, College of Chemistry & Pharmacy, Northwest A&F University, Xianyang 712100, China; qjz@nwafu.edu.cn

**Keywords:** *Ophiocordyceps sinensis*, *Thitarodes xiaojinensis*, codon usage bias, natural selection, mutation pressure, optimal codons

## Abstract

*Ophiocordyceps sinensis*, a fungus parasitic on insects, attracts interest due to its unique medicinal applications and complex ecological interactions. The potential relationship between codon usage bias and the parasitic relationship between *O. sinensis* and *Thitarodes xiaojinensis* was explored by analyzing the nuclear genomes and mitochondrial genomes of the two. The nuclear genomes showed contrasting preferences: *O. sinensis* sclerotium exhibited strong GC-ending codon bias (GC3 = 66.13%), while *T. xiaojinensis* favored AT-ending codons (GC3 = 49.14%). Conversely, both mitochondrial genomes displayed a strong preference (>70% AT) for AT-ending codons. Optimal codon analysis revealed 25 (GC-ending) in *O. sinensis* and 28 (GC-ending) in *T. xiaojinensis* nuclear genomes, with overlaps for Leu, Val, Ser, and Pro. The mitochondrial genomes had fewer optimal codons (16 and 13, AT-ending), showing limited overlap (Phe, Leu, Asp, Ser). Neutral plotting analysis, effective number of codons analysis, relative synonymous codon usage analysis, and parity bias analysis showed that the codon usage preferences of the nuclear genomes and mitochondrial genomes of *O. sinensis* and *T. xiaojinensis* are jointly affected by selection and mutation pressure. Natural selection predominates in nuclear genomes, while mutation pressure dominates in mitochondrial genomes. This provides new molecular insights into their host–parasite specificity.

## Simple Summary

*Ophiocordyceps sinensis*, a rare medicinal fungus endemic to the Qinghai–Tibet Plateau, exhibits a highly specific obligate parasitic relationship with its insect host, *Thitarodes xiaojinensis*. To investigate potential genetic underpinnings of this specificity, we analyzed codon usage bias in both the nuclear and mitochondrial genomes of the fungus and its host. The results indicate that codon usage in both organisms is shaped by both natural selection and mutational pressure. The nuclear genes are primarily driven by natural selection, whereas the mitochondrial genes are more influenced by mutational pressure. Notably, overlap in optimal codons for certain amino acids suggests possible co-adaptation at the gene-expression level. This study provides molecular evolutionary insights into the adaptation mechanisms of *O. sinensis* and its host, offering new perspectives on the evolution of obligate parasitism and a theoretical basis for future cultivation and conservation efforts.

## 1. Introduction

Codon usage bias refers to the phenomenon that synonymous codons show non-random distribution in the genome. This bias not only reflects the evolutionary trajectory of a species but may also affect the host adaptation ability of parasites by regulating the rate and accuracy of protein synthesis. In the genetic code, most amino acids are encoded by multiple codons, which usually differ only in the third position of the codon, namely synonymous codons [1,2]. Among the 64 codons, 61 codons encode 20 different amino acids; the remaining three serve as stop codons. Codon usage bias refers to the non-uniform usage of codons [3]. Various factors, such as expression level, gene length, and compositional bias, can all influence codon bias [4,5,6]. When the GC content of the genome changes, the changing trends and degrees of different codons will show significant differences. Specifically, the variation in the GC content of the codons themselves is the direct cause of the shift in codon usage bias within the genome. This process reveals important mechanisms in genome evolution. Changes in GC content usually stem from environmental stress, which directly affects codon composition. For example, in a high-GC environment, genomes tend to increase the usage frequency of GC-rich codons, such as GGC, CCC, and CGC, while reducing the proportion of AT-rich codons such as AAT, TTA, and ATA [7].

*O. sinensis* refers to the complex of the larval corpse and fungal stroma formed by the infection of the larvae of Hepialidae insects by *O. sinensis* [8]. It is a resource with extremely high ecological and economic value on the Qinghai–Tibet Plateau [3,9]. *O. sinensis* is also a traditional substance homologous to medicine and food; it is of great significance for improving public health and enhancing the quality of human life in the future [10,11].

Currently, there are extensive reports on the mitochondrial codon usage bias of Clavicipitaceae and Hepialidae. By analyzing the codon usage bias of *Samsoniella hepialid* and *Samsoniella yunnanensis*, it was found that there are significant differences in genomic characteristics and secondary metabolite biosynthetic gene clusters among fungi of different genera, different species, and different strains of the same species. What is more, the phylogenetic tree constructed by PKS (polyketide synthase) homologous sequences was highly similar to the multi-gene genetic distance tree, indicating that PKS homologous sequences can be used as molecular markers. By analyzing the mitochondria codons of *Thitarodes sejilaensis* from Sejila Mountain in Tibet, it was found that its mitochondria contain 13 protein-coding genes [12]. Most of these use the typical start codon ATN. However, *COI* (Cytochrome c Oxidase subunit I) and *ND1* (NADH dehydrogenase subunit 1) use the atypical start codons CGA and TTG; the top codons are mostly TAA. In addition, the researchers also studied the mitochondrial codon usage bias of *Thitarodes damxungensis* and *Thitarodes pui*. They found that the mitochondrial genomes of *T. damxungensis* and *T. pui* had high AT contents (82.5% and 81.4%) and that the gene arrangement order was the same as that of ancestral insects. Analysis based on the K2P (Kimura two-parameter) genetic distance showed that *T. pui* was most closely related to *T. sejilaensis* and that *T. damxungensis* was most closely related to *Thitarodes yunnanensis* [13].

There are relatively few reports on the nuclear genomes of Ophiocordycipitaceae and Hepialidae. Therefore, *Beauveria bassiana* was selected for codon bias analysis. Researchers analyzed *B. bassiana* and found that *B. bassiana* does not have a strong codon usage bias [14]. Codon bias is affected by multiple factors, such as mutational pressure and natural selection, and the selection for translation efficiency plays an important role in shaping codon usage bias. The study on the codon usage bias of *Epichloë festucae* found that the codon usage bias of *E. festucae* is related to gene length, hydrophobicity, and aromaticity [15]. There is a significant positive correlation between gene length and codon usage bias. The GRAVY value and Aromo value are negatively correlated with codon usage bias. In addition, the researchers analyzed the first comparative study on the codon usage patterns of two Lepidoptera insects, *Ostrinia furnacalis* and *Bombyx mandarina* [16]. The results indicated week codon usage bias in both species, primarily driven by nucleotide composition constraints and mutational pressure. Moreover, a preference for codons ending with cytosine (C) was observed, potentially attributable to GC-biased mutation dynamics [17].

To date, most existing studies have focused on the analysis of codon usage bias in single species. However, few studies have reported on the obligate parasitism of fungi on insects; no reports have been found on the comprehensive analysis of the two organisms in the obligate parasitic relationship. In this study, the codons of the nuclear genes of *O. sinensis* and *T. xiaojinensis*, as well as the mitochondrial genes of *O. sinensis* and *T. xiaojinensis*, were used as data for neutral analysis, ENC analysis, RSCU analysis, and optimal codon analysis to investigate the codon usage preferences between the nuclear genomes and mitochondrial genomes of *O. sinensis* and *T. xiaojinensis*.

## 2. Materials and Methods

### 2.1. Sequence Processing

The sequences of the mitochondrial gene serial number NC_034659.1, and nuclear gene accession number GCA_042246355.1 of *O. sinensis*, as well as the mitochondrial gene serial number NC_028348.1 and nuclear gene accession number GCA_012934285.1 of *T. xiaojinensis* were obtained from the GenBank database (https://www.ncbi.nlm.nih.gov/genbank/, accessed on 10 May 2025), respectively. The following screenings were performed on the downloaded sequences: (1) Ensure that the beginning of the sequence is the start codon ATG and the end is the stop codon; (2) Delete all sequences with a length less than 300 dp; (3) Ensure that there are no internal stop codons and that the open reading frame is correct; and (4) Remove redundant sequences with high similarity (95% sequence identity).

### 2.2. Codon Bias Analysis

Systematic analysis of the codon usage bias of *T. xiaojinensis* and *O. sinensis* was carried out using CodonW 1.42 and MEGA 12 software, to measure, in particular, the base composition of the first and third positions of codons (T3s, C3s, A3s, G3s, CG3s) and the genomic GC content, the effective number of codons (ENC), and the relative synonymous codon usage (RSCU), thus systematically comparing the base composition characteristics and codon usage preference parameters of the two organisms.

### 2.3. Determination of Optimal Codons

RSCU is the ratio of the usage frequency of a certain synonymous codon to the expected frequency without preference: the larger the RSCU value, the stronger the codon usage preference. The maximum value of the RSCU of synonymous codons of *T. xiaojinensis* and *O. sinensis* was taken as the optimal translation codon for the corresponding amino acid. Similarly, the RSCU values of synonymous codons of *T. xiaojinensis* and *O. sinensis* were calculated to summarize the characteristics of their optimal codons.

### 2.4. Drafting of ENC-GC3

In this study, the ENC-GC3 scatterplot analysis method was used. A coordinate system was constructed with the GC3 content as the abscissa and the observed effective number of codons (ENCobs) as the ordinate. The standard curve of the expected effective number of codons (ENCexp) was drawn according to the calculation formula proposed [18]. The ENC frequency ratio (ENCratio) was calculated using the formula (ENCexp − ENCobs)/ENCobs. Then, the distribution frequency map of each gene was constructed. This method quantifies the deviation between the expected and observed values to intuitively show the degree of codon usage bias.

The ENC, as a core indicator for measuring gene translation efficiency, has a numerical range with clear biological significance: when ENC = 20, it indicates complete codon bias (each amino acid uses only a single codon), while ENC = 61 reflects complete randomness (codons are used uniformly). This study systematically analyzed the ENCobs and ENCexp data of all genes and revealed the rule that lower values indicate stronger codon preference, while higher values tend towards random selection. This quantitative relationship provides an important basis for analyzing the adaptive evolution of species.

### 2.5. The Analysis of Parity Preference (PR2-Plot)

In this study, Microsoft Office Excel software was used to draw scatterplots. Analysis of the parity preference of codons is a scatterplot drawn based on the parity rule 2 (PR2), with A3/(A3 + T3) as the ordinate and G3/(G3 + C3) as the abscissa. When there are base mutations or selection biases between the two strands of DNA, the coordinate values of A3/(A3 + T3) and G3/(G3 + C3) will deviate from the central position of 0.5.

The core principle is to obtain AT-bias and GC-bias by evaluating and calculating the A/T/G/C content (A3S, T3S, G3S, C3S) of the third base of the codon. When the base substitution rates of the two DNA strands are balanced (for example, the rate of A → T on the W strand is equal to the rate of T → A on the complementary strand), the AT/GC content shows a symmetrical distribution; the graphical presentation uses a two-dimensional coordinate system, with AT-bias as the vertical axis and GC-bias as the horizontal axis. Data points close to the origin (0, 0) reflect mutation dominance, while deviation from the center indicates the effect of natural selection.

### 2.6. Neutral Analysis

Codon neutrality analysis was performed by plotting a scatterplot with GC3 as the abscissa and CG12 as the ordinate, and then by conducting linear regression fitting on it. If the slope of the fitted line approaches 0, it indicates a low correlation between GC3 and GC12. In this case, the codon usage bias is mainly driven by natural selection. Conversely, if the slope significantly deviates from 0, it reflects that the base mutation pressure has a stronger influence on codon bias. This analytical method effectively distinguishes the weight of the effects of natural selection and mutational pressure on codon evolution by quantifying the change in regression slope.

## 3. Results

### 3.1. Analysis of Base Composition of Genomic Codons in O. sinensis and T. xiaojinensis

**Figure 1:**
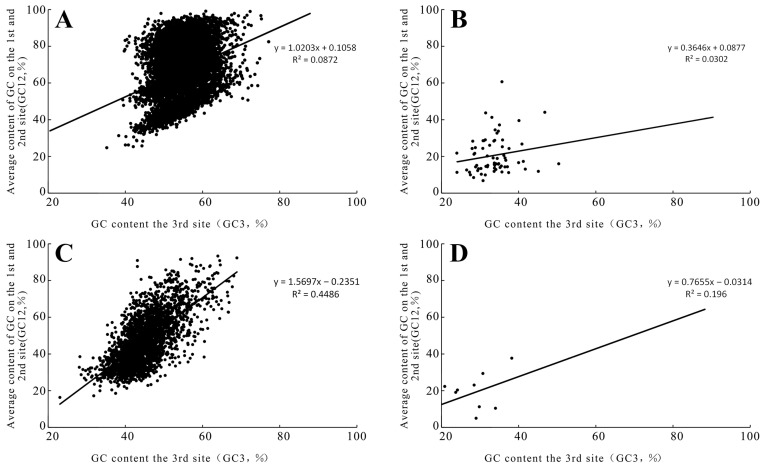
Neutral analysis (GC12 vs. GC3) of *Ophiocordyceps sinensis* and *Thitarodes xiaojinensis*: (**A**) neutral analysis of the nuclear genome of *Ophiocordyceps sinensis*; (**B**) neutral analysis of the mitochondrial genome of *Ophiocordyceps sinensis*; (**C**) neutral analysis of the nuclear genome of *Thitarodes xiaojinensis*; and (**D**) neutral analysis of the mitochondrial genome of *Thitarodes xiaojinensis*.

After screening, CDS sequences of 9485 nuclear genes and 69 mitochondrial genes were obtained from *O. sinensis*. Their average GC contents were 62.40% and 29.20%, respectively. The GC content of nuclear sequences of *O. sinensis* ranged from 81.60% to 27.20%; the GC content of sequence from the mitochondrial genomes of *O. sinensis* ranged from 46.90% to 20.30% (Table 1). The average GC contents at the three positions of codons in the nuclear genome were 61.17% (GC1), 47.15% (GC2), and 66.13% (GC3), respectively. The distribution range of GC12 was 42.00–75.00%. The average GC contents at the three positions of codons in its mitochondrial genome were 35.38% (GC1), 31.70% (GC2), and 20.99% (GC3), respectively. The distribution range of GC12 was 24.00–50.31% (Figure 1).

A total of 2870 mitochondrial and nuclear genomic sequences and 10 CDS sequences of the mitochondrial genome exist in *T. xiaojinensis*. The average GC contents are 46.43% and 20.97%, respectively. The GC content of each nuclear gene sequence in *T. xiaojinensis* ranges from 76.80% to 21.90%; the GC content of each mitochondrial sequence in *T. xiaojinensis* ranges from 12.8% to 28.1%. The average GC contents at the three positions of codons in the nuclear genome are 51.01% (GC1), 39.15% (GC2), and 49.14% (GC3), respectively. The distribution range of GC12 is from 22.60% to 84.65%. The average GC contents at the three positions of codons in its mitochondrial genome are 20.97% (GC1), 29.18% (GC2), and 19.26% (GC3), respectively; the distribution range of GC12 is from 17.42% to 38.23% (Figure 1).

### 3.2. Neutral Analysis of the Genomes of O. sinensis and T. xiaojinensis

The neutral analysis mainly revealed the correlation between GC12 and GC3 (Figure 1). There was a positive correlation between the nuclear genes of *O. sinensis* sclerotium (r = 0.08, *p* < 0.01) and its mitochondria (r = 0.03, *p* < 0.01); there was also a positive correlation between the nuclear genes of *T. xiaojinensis* (r = 0.44, *p* < 0.01) and its mitochondrial genome (r = 0.19, *p* < 0.01). This indicates that both the nuclear genomes and mitochondrial genomes of *O. sinensis* and *T. xiaojinensis* are affected by mutation pressure.

### 3.3. Frequency Distribution of Effective Codons of O. sinensis and T. xiaojinensis

**Figure 2:**
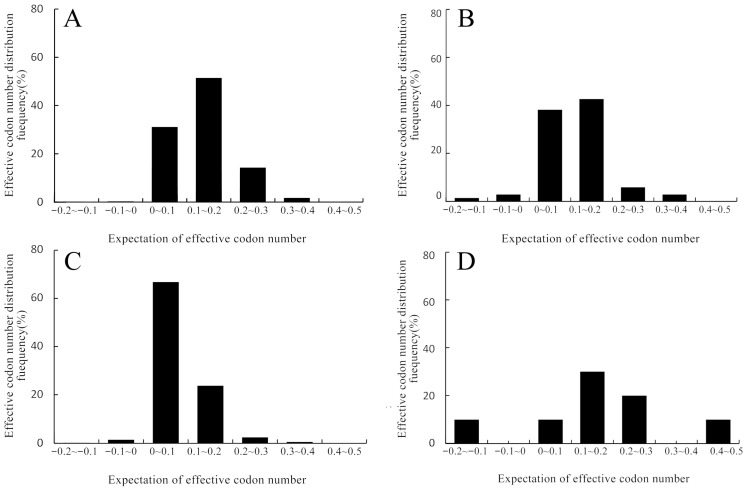
Frequency distribution of effective codons (ENC) in the genomes of *Ophiocordyceps sinensis* and *Thitarodes xiaojinensis*: (**A**) frequency analysis of the nuclear genome of *Ophiocordyceps sinensis*; (**B**) frequency analysis of the mitochondrial genome of *Ophiocordyceps sinensis*; (**C**) frequency analysis of the nuclear genome of *Thitarodes xiaojinensis*; and (**D**) frequency analysis of the mitochondrial genome of *Thitarodes xiaojinensis*.

The ENC ranges of the nuclear genome and mitochondrial genome of *O. sinensis* were 24.4–61.00 and 29.81–61.00, respectively; their average values were 47.72 and 41.46, respectively. Among the 9484 genes in the nuclear genome, only 967 genes showed a high codon preference (ENC < 35). Among the 69 genes in the mitochondrial genome, only 13 genes showed a high codon preference. Therefore, the codon usage preference of *O. sinensis* is weak. The ranges of the effective number of codons (ENC) in the nuclear genome and mitochondrial genome of *T. xiaojinensis* were 27.62–61.00 and 27.10–60.11, respectively; their average values were 54.27 and 40.99, respectively. Among the 2869 genes in the nuclear genome of *T. xiaojinensis*, only 15 genes showed a high codon usage bias (ENC < 35); among the 11 genes in the mitochondrial genome, only 3 genes showed a high codon usage bias. Therefore, the codon usage bias of *T. xiaojinensis* is also weak. The usage frequency of most effective codons of genes was distributed between 0 and 0.1, indicating that the observed values of most effective codons are lower than the expected values (Figure 2).

### 3.4. Correlation Analysis Between the Effective Number of Codons (ENC) and the Synonymous Codon Usage at the Third Position (GC3s) of O. sinensis and T. xiaojinensis

**Figure 3:**
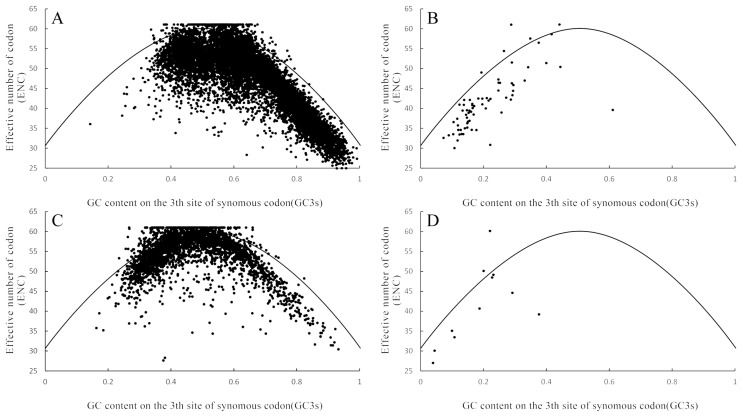
Plotting analysis of the effective number of codons (ENC) in the genomes of *Ophiocordyceps sinensis* and *Thitarodes xiaojinensis* to analyze the correlation between ENC and GC3s: (**A**) frequency analysis of the nuclear genome of *Ophiocordyceps sinensis*; (**B**) frequency analysis of the mitochondrial genome of *Ophiocordyceps sinensis*; (**C**) frequency analysis of the nuclear genome of *Thitarodes xiaojinensis*; and (**D**) frequency analysis of the mitochondrial genome of *Thitarodes xiaojinensis*.

Normally, ENC plotting analysis is used to explore the influence of GC3s on the codon usage pattern of the genome. If the effective number of codons (ENC) of a gene lies on the expected curve, it indicates that there is no codon usage bias in the gene. In this study, the ENC values of most genes were lower than the expected values and located at the lower right of the expected curve (Figure 3). Apart from the influence of other factors, mutational pressure is also an important factor affecting codon usage bias.

### 3.5. Correlation Analysis of Each Codon Index in the Genomes of O. sinensis and T. xiaojinensis

To determine the correlation between the relative codon usage preferences and nucleotide compositions of the genomes of *O. sinensis* and *T. xiaojinensis*, multivariate correlation analysis was used in this study to determine the interrelationship between codon usage preferences and hydrophobicity and aromaticity. The results showed that in the nuclear genome and mitochondrial genome of *O. sinensis*, there were extremely significant correlations between GC3s and Gravy, Aromo values (*p* < 0.01). In the nuclear genome of *T. xiaojinensis*, there were extremely significant correlations between GC3s and Gravy, Aromo values (*p* < 0.01), and there was an extremely significant correlation between the GC3s and Gravy value in the mitochondrial genome (*p* < 0.01).

The ENC values of the nuclear genome of *O. sinensis* (Table 2) were extremely significantly negatively correlated with the Gravy (r = −0.337, *p* < 0.01) and Aromo (r = −0.280, *p* < 0.01) values. In the mitochondrial genome of *O. sinensis* (Table 3), the ENC value was significantly correlated with Gravy (r = 0.270, *p* < 0.05). The ENC value of the nuclear genome of *T. xiaojinensis* (Table 4) was extremely significantly positively correlated with the Aromo value (r = 0.075, *p* < 0.05); the ENC value of the mitochondrial genome of *T. xiaojinensis* (Table 5) was extremely significantly positively correlated with the Aromo value (r = 0.931, *p* < 0.05). Meanwhile, the ENC value of the nuclear genome of *O. sinensis* (Table 2) showed a highly significant negative correlation with the first axis (r = −0.604, *p* < 0.05) and second axis (r = −0.458, *p* < 0.05), and a highly significant negative correlation with GC3s (r = −0.789, *p* < 0.01). The ENC value of the mitochondrial genome of *O. sinensis* (Table 3) showed a highly significant positive correlation with GC3s (r = 0.396, *p* < 0.01). The ENC value of the nuclear genome of *T. xiaojinensis* (Table 4) was positively correlated with the first axis (r = 0.135, *p* < 0.01) and second axis (r = 0.105, *p* < 0.01) and showed a highly significant negative correlation with GC3s (r = −0.221, *p* < 0.01). The ENC value of the mitochondrial genome of *T. xiaojinensis* (Table 5) showed a highly significant positive correlation with GC3s (r = 0.722, *p* < 0.01).

### 3.6. Relative Synonymous Codon Usage Frequency

**Figure 4:**
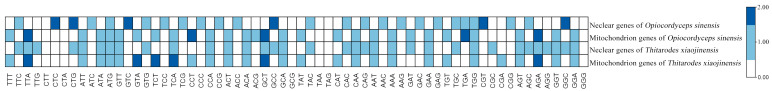
Heat map of amino acids and RSCU of *Ophiocordyceps sinensis* and *Thitarodes xiaojinensis.* Note: The optimal codons (ΔRSCU < 1) of *Ophiocordyceps sinensis* and *Thitarodes xiaojinensis* are marked in white, those with (1 < ΔRSCU < 2) are marked in light blue, and those with (ΔRSCU > 2) are marked in dark blue.

Through calculation, the usage times and frequencies (RSCU) of synonymous codons in the nuclear genome of *O. sinensis* were obtained (Figure 4). In both nuclear genes and mitochondrial genes, *O. sinensis* and *T. xiaojinensis* have some common frequently used codons, such as TTA, GCT, TCA, AGA, etc. Although there are common high-frequency codons, there are still certain differences in the specific usage frequencies and the usage of other codons. Especially in mitochondrial genes, there are obvious differences in codon usage preferences between the two. The frequencies of base codons ending with T and A are relatively high, and the RSCU values are greater than 1 (indicated by light blue), which are the codons preferentially used in the genomes of *O. sinensis* and *T. xiaojinensis*. In contrast, for C and G, the RSCU values are all less than 1, which are the codons with lower usage frequencies and are avoided by both.

### 3.7. Analysis of Parity Preference

**Figure 5:**
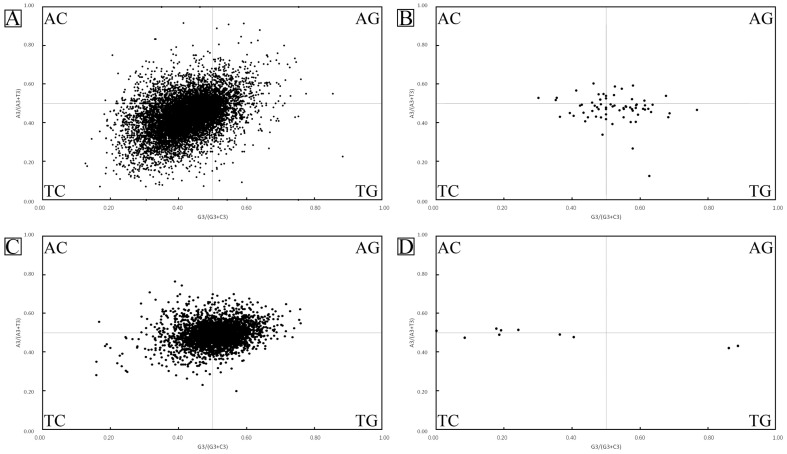
Nucleotide parity bias of nuclear and mitochondrial genes in *Ophiocordyceps sinensis* and *Thitarodes xiaojinensis*: (**A**) analysis of parity preference of nuclear genes in *Ophiocordyceps sinensis*; (**B**) analysis of parity preference of mitochondrial genes in *Ophiocordyceps sinensis*; (**C**) analysis of parity preference of nuclear genes in *Thitarodes xiaojinensis*; and (**D**) analysis of parity preference of mitochondrial genes in *Thitarodes xiaojinensis*.

Odd–even preference is mainly used to analyze the relationship between pyrimidines and purines at the third position of codons (Figure 5). In the sclerotium genes and mitochondrial genes of *O. sinensis*, the content of pyrimidines (C + T) at the third position is greater than that of purines (A + G); in *T. xiaojinensis,* the content of pyrimidines (C + T) at the third position is approximately equal to that of purines (A + G). Generally, it is considered that when G and C (or A and T) are proportionally distributed at the third position of codons, it indicates that the codon usage bias is affected by mutation pressure; if G and C (or A and T) are disproportionately distributed at the third position of codons; it indicates that the codon usage bias is affected by natural selection pressure.

Therefore, based on the parity analysis, the codon usage bias of the genes of *O. sinensis* may be affected by natural selection pressure, while the codon usage bias of the genes of *T. xiaojinensis* may be affected by mutation pressure.

### 3.8. Optimal Codon Analysis

In this study, the ΔRSCU method was used to identify the optimal codons. By calculating the optimal codon tables for the entire genome, the tables show the synonymous codons encoding each amino acid and the corresponding RSCU values of “high expression” and “low expression” for each synonymous codon. The ΔRSCU values were obtained. In the nuclear genome of *O. sinensis*, 25 optimal codons and 16 optimal codons were detected in the mitochondrial genome. Among these, in the nuclear genome, 10 codons end with G and 15 end with C; in the mitochondrial genome, 9 codons end with T and 7 end with A. This indicates that these are related to the GC content at the third position of the codons in the nuclear genome; the codons in the mitochondrial genome may be related to the AT content at the third position. In the nuclear genome of *T. xiaojinensis*, there are 28 optimal codons, and 13 optimal codons in the mitochondrial genome. Among these, in the nuclear genome, 12 codons end with G and 16 end with C; in the mitochondrial genome, 10 codons end with T and 3 end with A (Table 6).

In addition, there are two optimal codons for Leucine (Leu), Valine (Val), Serine (Ser), and Proline (Pro) in the nuclear genome of *O. sinensis*. There are also two optimal codons for Leucine (Leu), Valine (Val), Serine (Ser), Proline (Pro), Threonine (Thr), Alanine (Ala), and Arginine (Arg) in the nuclear genome of *T. xiaojinensis*.

## 4. Discussion

By analyzing the codon usage biases of *O. sinensis* and *T. xiaojinensis*, it can be shown that the nuclear genes of *O. sinensis* may be mainly affected by natural selection, while the nuclear genes of *T. xiaojinensis* may be affected by mutational pressure. Moreover, the mitochondrial genes of both *O. sinensis* and *T. xiaojinensis* are mainly affected by mutational pressure.

The analysis revealed that the nuclear genome of *O. sinensis* exhibits 25 optimal codons, with 10 ending in G and 15 ending in C. The nuclear genome of *T. xiaojinensis* contains 28 optimal codons, with 12 ending in G and 16 ending in C. Therefore, it is inferred that most of the codons in the nuclear genomes of *O. sinensis* and *T. xiaojinensis* tend to end with G or C, which is mainly affected by natural selection. In the analysis of codon usage bias of fungi, such as *Amanita*, *Volvariella volvacea*, and *Pleurotus* [19,20], researchers found that the nuclear genes of these species all prefer codons ending with C or G, which is similar to the research results of the nuclear genes of *O. sinensis* [21,22]. The analysis shows that the nuclear genes of Lepidoptera insects, such as *O. furnacalis* and *B. mandarina*, prefer to end with C or G, which is similar to the results of this study [16]. In studies on *Heliconius melpomene*, *Danaus plexippus*, *Callosamia cecrops*, *Parides sennea*, and *Drosophila melanogaster* [23], it was found that the codon preferences of nuclear genes in these species all favored codons ending with A or T. This is inconsistent with the results of the codon preference analysis of the nuclear genome of *T. xiaojinensis*. This could be attributed to butterflies and hepialid moths belonging to distinct families within the order Lepidoptera; for instance, butterflies typically fall under the superfamily Papilionoidea, while hepialid moths belong to the family Hepialidae. The nuclear genes of fungi in yeasts, such as *Saccharomyces cerevisiae* and *Schizosaccharomyces pombe*, prefer to end with A or T, which is inconsistent with the research results of the nuclear genes of *O. sinensis*. This may be related to the genetic evolution or environmental adaptability of these species [24].

In the mitochondrial genome of *O. sinensis*, 16 optimal codons were identified, comprising seven ending with A and nine ending with T. In contrast, the mitochondrial genome of *T. xiaojinensis* contains 13 optimal codons, with 3 ending with A and 10 ending with T. Consequently, it is concluded that mitochondrial genomic codon usage in *O. sinensis* and *T. xiaojinensis* predominantly terminates with A or T, and is primarily driven by natural selection. Analysis of mitochondrial codon usage bias in *Amanita sinensis*, *Amanita muscaria* and *Amanita phalloides* [25] and *S. hepialid* revealed a consistent preference for codons ending with A or T, aligning with findings from studies on the mitochondrial genome of *O. sinensis*. Analysis of mitochondrial codon usage bias in *Kallima inachus*, *Argynnis hyperbius* and *Brachmia macroscopa*, *Potanthus flavus* revealed a consistent preference for codons ending with A or T, aligning with the mitochondrial codon preference observed in *T. xiaojinensis* [26,27]. Analysis of mitochondrial codon usage bias in diverse higher plants—including *Medicago polymorpha*, *Medicago sativa*, and *Medicago truncatula*—revealed that despite phylogenetic divergence across plant families, their mitochondrial preferred codons predominantly terminate with A or T [28,29]. This finding aligns with results from the present study and may reflect conserved evolutionary traits stemming from the monophyletic origin of mitochondria in ancestral microorganisms [30].

ENC analysis demonstrated that the effective number of codons (ENC) for nuclear genes ranged from 24.4 to 61.0 in *O. sinensis* sclerotia and 26.4 to 61.0 in *T. xiaojinensis*, whereas mitochondrial genes exhibited distinct distributions: *O. sinensis* mitochondria spanned 29.8–61.0, while *T. xiaojinensis* mitochondria showed a markedly narrower ENC range (27.1–49.13). Comprehensive codon usage analysis employing CodonW and MEGA integrated three complementary approaches: ENC-GC3s correlation plots, parity rule 2 (PR2) analysis, and neutrality tests to characterize usage patterns and elucidate evolutionary mechanisms in both organisms.

Codon bias is usually affected to varying degrees by multiple factors such as gene length, gene expression level, genomic base composition, transcription, and translation. The selection–mutation–drift model suggests that, among numerous influencing factors, base mutation pressure and natural selection pressure are the two most important ones. The codon usage biases of different fungal genomes also vary. The nuclear genome tends to be affected by selection pressure, while the mitochondrial genome is more strongly influenced by base mutations, which is consistent with the results of this study [31]. Mutational selection pressure is considered to be the most significant factor contributing to codon asymmetry. Through neutral plotting analysis of the nuclear and mitochondrial genomes of *O. sinensis* (Figure 1), it was found that the codons of *O. sinensis* are subject to directional mutational pressure under natural selection; similar conclusions were drawn in the study of *B. bassiana* [32,33]. This result is the same as that of other fungi living a parasitic life. Their codons are also affected by strong directional mutation pressure. Therefore, it is considered that the directional pressure on the codons of *O. sinensis* may be related to its parasitic life, and that the pressure may come from its host [34].

Data show that, for both *O. sinensis* and *T. xiaojinensis*, the effective number of codon (ENC) values of their nuclear genes and mitochondrial genes are greater than 35, indicating that the codon usage bias of their nuclear genomes and mitochondrial genomes is weak [35,36]. Within the *O. sinensis*–host insect system, this weak bias may arise from the parasitic life history: as an obligate parasitic fungus, *O. sinensis* genomic evolution is influenced by co-evolution with its host, reducing gene expression constraints. Concurrently, the host insect *T. xiaojinensis* genome likely maintains genetic diversity through selection mechanisms to adapt to high-altitude environments [37].

Furthermore, *O. sinensis* exhibits a relatively long lifecycle among cordyceps fungi; its ecological adaptation strategy sacrifices reproductive speed for host specificity and survival advantages in extreme environments. Genome analysis of *O. sinensis* reveals a codon usage bias significantly favoring low-frequency codons. This bias optimizes gene translation efficiency, meets protein synthesis demands in low-temperature, high-altitude conditions, and aligns with co-evolutionary mechanisms involving the slow lifecycle and ecological strategy [38,39].

As an obligate parasite of *T. xiaojinensis*, *O. sinensis* can evade competitive microbial pressures. From a nuclear gene perspective, *O. sinensis* may enhance translation efficiency for host-cell regulation via codon bias optimization in specific genes such as those encoding parasitic effector proteins [40]. Conversely, weak codon bias in the nuclear genes of *T. xiaojinensis* reflects diverse mutation strategies responding to parasitic pressures [41]. Regarding mitochondrial gene interactions, *O. sinensis* retains strong codon bias in mitochondrial genomes (COX1 and ND4 genes), potentially linked to its reliance on host mitochondrial energy metabolism [42]. In contrast, *T. xiaojinensis* mitochondrial genome shows low-preference patterns, adapting to metabolic plasticity during larval stages in hypoxic, low-temperature environments [43]. Notably, complementary codon pairing occurs between the two species—for instance, *O. sinensis* prefers NNT codons while *T. xiaojinensis* favors NNA anticodons in the ND2 gene. This suggests a cross-species coordinated regulatory mechanism for mitochondrial gene expression, possibly serving as a key molecular interface in obligate parasitic system evolution [44,45]. Collectively, the dual co-evolutionary pathways of nuclear and mitochondrial genomes maintain a dynamic balance between *O. sinensis* breaching host immune barriers and host-restricted metabolic resource allocation [46].

An obligate parasitic relationship exists between *O. sinecsis* and *T. xiaojinensis*; this host specificity is manifested as the extreme specificity of the former only being able to infect the larvae of the latter, which is in sharp contrast to the generalist parasitism that can infect hundreds of hosts [47]. This difference essentially stems from the constraints of the “host unit”—jointly shaped by the host niche such as the high-altitude habitat of ghost moth larvae, and the co-evolutionary history of the parasitic system [48]. Although host specificity is a common pattern in parasitic systems, there are still significant gaps in the research on the molecular mechanisms of obligate parasitic taxa, as an obligate parasitic fungus with both medicinal value and ecological representativeness, *O. sinensis* and its interaction system with its key host, *T. xiaojinensis*, provide an ideal model for exploring the adaptive functions of codon bias [49]. By focusing on the differences in codon usage preferences between the two, this study aims to decipher the molecular adaptation mechanisms of obligate hosts and host organisms at the levels of gene expression regulation and protein synthesis, providing a theoretical basis for revealing the evolutionary driving forces of the obligate parasitic interaction relationship [50,51].

Based on existing research, we propose a new hypothesis: codon preference is jointly influenced by natural selection, mutational pressure, and host ecological adaptability [52]. For example, in obligate parasitic fungi, such as *O. sinensis*, this fungus forms a coevolutionary mechanism with its host to cope with survival pressures [53]. Future research can explore the dynamic change patterns under different ecological gradients, particularly adaptations to extreme environments. Additionally, single-cell sequencing technology can be used to analyze the molecular coevolutionary trajectories of the host–parasite system, which will provide new perspectives for understanding the evolutionary dynamics of obligate parasitic relationships [54].

## 5. Conclusions

This study systematically analyzed the codon usage bias in the nuclear and mitochondrial genomes of *O. sinensis* and its host insect, *T. xiaojinensis*, revealing significant differences in codon preference between the two and their evolutionary driving forces. The results showed that the nuclear genome of *O. sinensis* exhibited a strong GC3 preference and a codon usage pattern dominated by natural selection, while the nuclear genes of *T. xiaojinensis* were more significantly affected by mutational pressure. In the mitochondrial genomes, both showed high AT content and were mainly dominated by mutational pressure [55]. In addition, although there was some overlap in the selection of optimal codons between the two organisms, the overall biases were different, suggesting that they have formed unique molecular adaptation mechanisms during their long-term obligate parasitic relationship.

## Figures and Tables

**Table 1:** Basic compositional information, ENC, Gravy, and Aromo of codons in *Ophiocordyceps sinensis* and *Thitarodes xiaojinensis*.

Class	Genes	Codons	GC (%)	GC1 (%)	GC2 (%)	GC3 (%)	T3s (%)	C3s (%)	A3s (%)	G3s (%)	Gravy	Aromo	ENC	CAI
Nuclear genes of *Ophiocordyceps sinensis*	9484	4,637,889	62.4	61.74	47.15	66.13	25.5	88.84	20.33	65.31	−0.59	0.05	47.12	0.05
Mitochondrial genes of *Ophiocordyceps sinensis*	68	18,651	29.21	35.39	31.7	21.01	84.43	18.79	76.45	20.32	0.01	0.12	41.47	0.17
Nuclear genes of *Thitarodes xiaojinensis*	2868	1,356,889	46.43	51.01	39.15	49.14	51.42	48.55	50.28	49.73	−0.35	0.08	57.27	0.21
Mitochondrial genes of *Thitarodes xiaojinensis*	11	3240	20.97	20.9	29.18	19.26	95.16	10.04	30.04	4.74	0.74	0.23	40.99	0.12

**Table 2:** Correlation analysis of various parameters of the nuclear genome of *Ophiocordyceps sinensis*.

	GC	GC1	GC2	GC3	GC3s	A3s	T3s	C3s	G3s	Gravy	Aromo	ENC	CAI	Axis1
GC1	0.640 **													
GC2	0.517 **	0.191 **												
GC3	0.749 **	0.229 **	−0.022 *											
GC3s	0.413 **	0.267 **	0.194 **	0.407 **										
A3s	−0.394 **	−0.251 **	−0.207 **	−0.385 **	−0.943 **									
T3s	−0.458 **	−0.271 **	−0.185 **	−0.440 **	−0.870 **	0.677 **								
C3s	0.294 **	0.185 **	0.117 **	0.330 **	0.854 **	−0.836 **	−0.636 **							
G3s	0.273 **	0.159 **	0.011	0.311 **	0.198 **	−0.113 **	−0.270 **	−0.067 **						
Gravy	−0.022 *	−0.038 **	0.042 **	0.026 **	0.580 **	−0.624 **	−0.419 **	0.531 **	−0.125 **					
Aromo	−0.257 **	−0.245 **	−0.151 **	−0.082 **	0.472 **	−0.462 **	−0.337 **	0.472 **	−0.095 **	0.557 **				
ENC	−0.528 **	−0.284 **	−0.083 **	−0.616 **	−0.789 **	0.745 **	0.682 **	−0.798 **	−0.135 **	−0.377 **	−0.280 **			
CAI	0.045 **	0.068 **	−0.014	0.129 **	0.698 **	−0.745 **	−0.384 **	0.827 **	−0.024*	0.487 **	0.472 **	−0.604 **		
Axis1	−0.135 **	0.051 **	−0.116 **	−0.062 **	0.513 **	−0.536 **	−0.205 **	0.712 **	0.078 **	0.408 **	0.400 **	−0.458 **	0.781 **	
Axis2	−0.197 **	−0.194 **	−0.052 **	−0.092 **	0.564 **	−0.503 **	−0.587 **	0.308 **	−0.039 **	0.545 **	0.501 **	−0.204 **	0.308 **	0.024 *

Note: **. The correlation is significant at the 0.01 level *. The correlation is significant at the 0.05 level.

**Table 3:** Correlation analysis of various parameters of the mitochondrial genome of *Ophiocordyceps sinensis*.

	GC	GC1	GC2	GC3	GC3s	A3s	T3s	C3s	G3s	Gravy	Aromo	ENC	CAI	Axis1
GC1	0.790 **													
GC2	0.489 **	0.158												
GC3	0.584 **	0.437 **	−0.208											
GC3s	0.584 **	0.437 **	−0.208	0.998 **										
A3s	−0.574 **	−0.498 **	0.065	−0.820 **	−0.820 **									
T3s	−0.482 **	−0.316 **	0.085	−0.751 **	−0.751 **	0.339 **								
C3s	0.521 **	0.481 **	−0.277 *	0.918 **	0.918 **	−0.786 **	−0.687 **							
G3s	0.317 **	0.022	−0.026	0.632 **	0.632 **	−0.426 **	−0.469 **	0.284 *						
Gravy	−0.061	0.048	−0.397 **	0.407 **	0.407 **	−0.337 **	−0.537 **	0.432 **	0.132					
Aromo	−0.141	−0.050	−0.336 **	0.253 *	0.253 *	−0.313 **	−0.038	0.329 **	−0.006	0.422 **				
ENC	0.455 **	0.185	−0.011	0.722 **	0.722 **	−0.516 **	−0.623 **	0.489 **	0.802 **	0.270 *	0.063			
CAI	0.335 **	0.557 **	−0.218	0.396 **	0.396 **	−0.569 **	0.032	0.559 **	−0.189	−0.050	0.222	−0.071		
Axis1	0.037	0.026	−0.266 *	0.195	0.195	0.037	−0.249 *	0.202	0.086	0.036	0.049	0.062	0.127	
Axis2	−0.017	0.004	−0.094	0.044	0.044	0.000	−0.030	0.019	0.097	0.067	0.015	0.035	−0.082	0.024 *

Note: **. The correlation is significant at the 0.01 level *. The correlation is significant at the 0.05 level.

**Table 4:** Correlation analysis of various parameters of the nuclear genome of *Thitarodes xiaojinensis*.

	GC	GC1	GC2	GC3	GC3s	A3s	T3s	C3s	G3s	Gravy	Aromo	ENC	CAI	Axis1
GC1	0.831 **													
GC2	0.719 **	0.495 **												
GC3	0.925 **	0.658 **	0.491 **											
GC3s	0.926 **	0.658 **	0.491 **	0.980 **										
A3s	−0.900 **	−0.658 **	−0.534 **	−0.935 **	−0.935 **									
T3s	−0.871 **	−0.604 **	−0.506 **	−0.927 **	−0.927 **	0.765 **								
C3s	0.837 **	0.566 **	0.437 **	0.924 **	0.924 **	−0.836 **	−0.867 **							
G3s	0.722 **	0.520 **	0.236 **	0.847 **	0.847 **	−0.796 **	−0.745 **	0.619 **						
Gravy	0.058 **	−0.061 **	0.071 **	0.087 **	0.087 **	−0.177 **	−0.123 **	0.069 **	−0.031					
Aromo	−0.229 **	−0.389 **	−0.206 **	−0.101 **	−0.101 **	0.088 **	0.115 **	−0.009	−0.141 **	0.415 **				
ENC	−0.218 **	−0.149 **	−0.154 **	−0.221 **	−0.221 **	0.177 **	0.216 **	−0.238 **	−0.085 **	−0.004	0.075 **			
CAI	0.481 **	0.420 **	0.140 **	0.536 **	0.537 **	−0.538 **	−0.367 **	0.639 **	0.357 **	−0.128 **	−0.043 *	−0.191 **		
Axis1	−0.741 **	−0.623 **	−0.818 **	−0.540 **	−0.541 **	0.606 **	0.548 **	−0.483 **	−0.271 **	−0.0429 **	−0.058 **	0.135 **	−0.0126 **	
Axis2	−0.363 **	−0.537 **	−0.264 **	−0.221 **	−0.221 **	0.163 **	0.217 **	−0.132 **	−0.255 **	0.732 **	0.734 **	0.105 **	−0.179 **	−0.010

Note: **. The correlation is significant at the 0.01 level *. The correlation is significant at the 0.05 level.

**Table 5:** Correlation analysis of various parameters of the mitochondrial genome of *Thitarodes xiaojinensis*.

	GC	GC1	GC2	GC3	GC3s	A3s	T3s	C3s	G3s	Gravy	Aromo	ENC	CAI	Axis1
GC1	−0.230													
GC2	−0.336	0.937 **												
GC3	−0.101	0.382	0.117											
GC3s	0.444	−0.090	0.013	−0.665 *										
A3s	−0.311	0.048	−0.054	0.701 *	−0.954 **									
T3s	−0.434	−0.079	0.092	−0.528	0.243	−0.483								
C3s	0.437	0.129	0.220	−0.548	0.909 **	−0.876 **	0.261							
G3s	0.268	−0.297	−0.179	−0.698 *	0.890 **	−0.870 **	0.296	0.627						
Gravy	−0.395	−0.185	−0.241	0.338	−0.586	0.579	−0.240	−0.817 **	−0.238					
Aromo	−0.111	−0.309	−0.136	−0.779 **	0.718 *	−0.835 **	0.778 **	0.589	0.785 **	−0.330				
ENC	−0.057	−0.407	−0.190	−0.856 **	0.592	−0.729 *	0.840 **	0.507	0.646 *	−0.379	0.931 **			
CAI	0.361	−0.046	0.096	−0.705 *	0.842 **	−0.906 **	0.567	0.871 **	0.668 *	−0.645 *	0.751 *	0.760 *		
Axis1	−0.274	−0.512	−0.517	0.094	−0.469	0.441	−0.051	−0.750 *	−0.077	0.909 **	−0.108	−0.074	−0.463	
Axis2	−0.198	−0.154	0.017	−0.691 *	0.750 *	−0.810 **	0.596	0.567	0.858 **	−0.259	0.917 **	0.756 *	0.602	−0.148

Note: **. The correlation is significant at the 0.01 level *. The correlation is significant at the 0.05 level.

**Table 6:** Determination of the optimal codons of *Ophiocordyceps sinensis* and *Thitarodes xiaojinensis*.

Amino Acid	Codon	Nuclear Gene of *Ophiocordyceps sinensis*	Mitochondrial Gene of *Ophiocordyceps sinensis*	Nuclear Gene of *Thitarodes xiaojinensis*	Mitochondrial Gene of *Thitarodes xiaojinensis*	Amino Acid	Codon	Nuclear Gene of *Ophiocordyceps sinensis*	Mitochondrial Gene of *Ophiocordyceps sinensis*	Nuclear Gene of *Thitarodes xiaojinensis*	Mitochondrial Gene of *Thitarodes xiaojinensis*
Phe	TTT		*		*	Ser	TCT		*		*
	TTC	*		*			TCC	*		*	
Leu	TTA		*		*		TCA				
	TTG						TCG	*		*	
	CTT					Pro	CCT		*		*
	CTC	*		*			CCC	*		*	
	CTA						CCA				
	CTG	*		*			CCG	*		*	
Ile	ATT				*	Thr	ACT				
	ATC	*		*			ACC	*		*	
	ATA		*				ACA		*		
Met	ATG						ACG	*		*	
Val	GTT					Ala	GCT				
	GTC	*		*			GCC	*		*	
	GTA		*				GCA				
	GTG	*		*			GCG			*	
Tyr	TAT		*		*	Trp	TGG				
	TAC	*		*		Arg	CGT				
Cys	TGT		*		*		CGC	*		*	
	TGC	*		*			CGA				
His	CAT				*		CGG			*	
	CAC	*		*		Ser	AGT		*		
Gln	CAA		*				AGC	*		*	
	CAG	*		*		Arg	AGA		*		
Asn	AAT		*		*		AGG	*			
	AAC	*		*		Gly	GGT		*		
Lys	AAA		*				GGC	*		*	
	AAG	*		*			GGA				
Asp	GAT		*		*		GGG			*	*
	GAC	*		*		TER	TGA				
Glu	GAA						TAA				
	GAG	*		*			TAG				

Note: * indicates the optimal codon.

## Data Availability

The genomic data of the nuclear and mitochondria of *O. sinensis* have been deposited in the international GenBank database. The mitochondrial genomic data of *T. xiaojinensis* have also been deposited in the international GenBank database.
